# Acquired tracheomalacia due to SARS‐CoV‐2 pneumonia

**DOI:** 10.1111/crj.13719

**Published:** 2023-12-20

**Authors:** David Espejo, Marta Zapata, Saliha Omari, Xavier Muñoz, Maria‐Jesús Cruz, Se‐COVID‐19 team, Jordi Andreu, Marta Arjona, Cristina Berastegui, Miriam Barrecheguren, José Cardoso, David Clofent, Almudena Felipe, Galo‐David Granados, Ma Ángeles Jiménez, Manuel López, Íñigo Ojanguren, Jeisson Osorio, Mercedes Pallero, María‐Florencia Pilia, Ma Antonia Ramon, Eva Ma Revilla, Christian Romero‐Mesones, Berta Sáez, Júlia Sampol, Eduardo Vélez, Ana Villar

**Affiliations:** ^1^ Pulmonology Service, Department of Medicine, Vall d'Hebron University Hospital Autonomous University of Barcelona Barcelona Spain; ^2^ CIBER of Respiratory Diseases (CIBERES) Barcelona Spain; ^3^ Department of Cell Biology, Physiology and Immunology Autonomous University of Barcelona Barcelona Spain

**Keywords:** COVID‐19, infection, inflammation

## Abstract

**Introduction:**

Several studies mentioned parenchymal findings after SARS‐CoV‐2 pneumonia, but few studies have mentioned alterations in the airways. The aim of this study was to estimate the prevalence of tracheomalacia and to analyse the clinical characteristics in a cohort of patients with SARS‐CoV‐2.

**Methods:**

The study population consisted of all patients with SARS‐CoV‐2 admitted a hospital serving a population of 500 000 inhabitants. Patients were visited between 2 and 6 months after hospital discharge. In this visit, all patients were subjected to an exhaustive clinical questionnaire and underwent clinical examination, pulmonary function tests and chest CT.

**Results:**

From February 2020 to August 2021, 1920 patients were included in the cohort and tracheomalacia was observed in 15 (0.8%) on expiratory HRCT imaging. All patients with tracheomalacia also presented ground glass opacities in the CT scan and 12 patients had airway sequelae.

**Conclusions:**

Tracheomalacia is an exceptional sequela of SARS‐CoV‐2 survivors.

To the Editor:

Several studies have found that SARS‐CoV‐2 pneumonia has many respiratory consequences, including clinical, functional and radiological sequelae.^1^ Radiologically, numerous parenchymal findings such as ground‐glass opacities, consolidations, reticulation patterns or pulmonary fibrosis have been recorded.^2^ Nevertheless, few studies have mentioned alterations in the airways: among the alterations reported, bronchiectasis is the most frequent.^3^, ^4^

Tracheomalacia is defined as a diffuse or segmental weakness of the trachea due to the loss of structural integrity of the cartilage. Clinical presentation of tracheomalacia is nonspecific and its most frequent symptoms are dyspnoea and cough. Classically, it has been defined as primary (congenital) or secondary (acquired). Airway inflammation is a secondary cause of acquired forms of tracheomalacia including airway infections, airway or lung malignancy, inhalation of chemical irritants and prolonged intubation or tracheostomy with mechanical ventilation.^5^, ^6^, ^7^

The aim of this study was to estimate the prevalence of tracheomalacia and to analyse the clinical characteristics in a cohort including all patients with SARS‐CoV‐2 pneumonia confirmed by PCR admitted to a reference centre in a hospital serving a population of 500 000 inhabitants at the beginning of the pandemic. To identify the respiratory sequelae, all patients were administered an exhaustive clinical questionnaire and underwent clinical examination, pulmonary function tests, and chest CT between 2 and 6 months after hospital discharge.

From February 2020 to August 2021, 1920 patients were included in the cohort. Tracheomalacia was observed in 15 (0.8%) on expiratory HRCT imaging. The median age of these patients was 60 years, and their baseline characteristics are summarized in Table 1. The treatment for SARS‐CoV‐2 infection in these patients was modified in the light of the new information obtained during the different waves of the pandemic. Several treatment protocols were applied including antiviral therapies as well as corticoids and empirical antibiotherapy with cephalosporins and quinolones. In this group, 10 patients received hydroxychloroquine, nine lopinavir/ritonavir, two tocilizumab, five dexamethasone and 13 azithromycin (some patients received several treatments at the same time). Regarding the symptoms identified on clinical examination, only three (20%) patients were asymptomatic; 10 (67%) had dyspnoea and two (13%) had cough. Table 1 also shows the findings reported at the follow‐up visit for pulmonary respiratory function and chest CT. In 14 patients, the tracheomalacia was diffuse, affecting the entire trachea. Of these, in six cases, there was clear associated bronchomalacia. In one patient, the malacia was segmental at the level of the upper third of the trachea. No tracheal stenosis was observed. In nine patients, tracheomalacia affected both at the membranous and cartilaginous level, and in six cases, the membranous part was only affected.^8^ Ground glass opacities in the CT scan were found in all patients with tracheomalacia. Six patients (40%) also presented other parenchymatous alterations such as septal thickening and reticulation. Twelve patients also had airway sequelae such as bronchiectasis, bronchiolitis or airway bronchial thickening.

**TABLE 1:** Baseline characteristics of 15 patients with tracheomalacia in thoracic HRCT.

Number of patients	15
Gender, male *n* (%)	8 (53)
Age, median (range)	65.5 (48–75)
Smokers, *n* (%)	4 (27)
Respiratory disease, *n* (%)	
Chronic obstructive pulmonary disease	1 (7)
Asthma	2 (13.3)
Obstructive sleep apnoea	2 (13.3)
None	10 (67)
Ventilatory support, *n* (%)	
Invasive ventilatory support	5 (33.3)
Non‐invasive ventilatory support	3 (20)
O_2_ venturi effect	2 (13.3)
No oxygen therapy or ventilation support	5 (33.3)
Radiology pattern, *n* (%)	
Unilobal pneumonia	5 (33)
Multilobar pneumonia	10(67)
Diffuse intersticial disease	0
Severity of SARS‐CoV‐2 pneumonia, *n* (%)^a^	
Mild	5 (33)
Severe	2 (13)
Very severe	0 (0)
Critical	8 (54)
Data from follow‐up visit	
Pulmonary respiratory function test, median (range)	
Forced vital capacity (FVC%)	89.2 (66–123)
Forced expiratory volume in the first second (FEV1%)	92.3 (38–131)
FVC/FEV1	83.3 (43–99)
Diffusing capacity for carbon monoxide (DLCO%)	64.7 (43–109)
HRCT tracheomalacia characteristics, *n* (%)	
Membranous part	6 (40)
Membranous and cartilaginous (both)	9 (60)
Cicatricial stenosis	0
Location:	
Segmental (upper third)	1 (7)
Diffuse	14 (93)
HRCT parenchymal sequelae, *n* (%)	
Ground glass opacities	15 (100)
Ground glass opacities and reticulation or septal thickening	6 (40)
HRCT airway tract sequelae, *n* (%)	
Bronchiectasis	9 (60)
Bronchial thickening	1 (7)
Bronchiolytis	2 (13)
Only tracheomalacia	3 (20)
Treatment, *n* (%)	
No treatment	8 (53)
LABA	2 (13)
LAMA	2 (13)
Inhaled cortocosteroids	2 (13)
Oral corticosteroids	2 (13)
CPAP^b^	1 (7)
Surgery^c^	0 (0)

Abbreviations: HRCT, high‐resolution computed tomography; LABA, long‐acting beta‐agonists; LAMA, long‐acting muscarinic antagonists; O_2_ Venturi effect, oxygen provided by Venturi system.

^a^ The severity of COVID‐19 was recorded based on the needs of oxygen and ventilatory support. Disease was defined as severe if the patient needed FiO2 < 40%, very severe if they needed FiO2 > 40%, and critical if they needed ventilatory support.

^b^ A patient was diagnosed with sleep apnea syndrome.

^c^ Referred to respiratory rehabilitation.

These results suggest that the probability of presenting tracheomalacia due to SARS CoV2 pneumonia is low. In our opinion, there are three possible explanations for the appearance of tracheomalacia in the SARS CoV‐2 survivor population. The first one is orotracheal intubation. Although the prevalence of primary or secondary tracheomalacia is unknown in the general population, its most common acquired cause is orotracheal intubation or tracheostomy. In these cases, tracheomalacia is related to increases in the respiratory airway pressure, oxygen toxicity and recurrent infections.^6^ Recently, Guarnieri et al.^9^ reported tracheomalacia in 8 of 151 patients with SARS‐CoV‐2 acute respiratory distress syndrome who required intubation or tracheostomy and mechanical ventilation. In the present series, however, intubation was only required in five patients.

Secondly, respiratory infections are a well‐known cause of tracheomalacia and were present in 67% of our patients. It should be highlighted that chronic infections are more strongly associated with tracheomalacia than acute presentations. However, in our experience, tracheomalacia is not an isolated finding, because all patients had parenchymal alterations and 80% had other airway sequelae, as shown in Table 1. Therefore, high levels of inflammation, such as those related with SARS CoV‐2 infection, may be responsible for these alterations. In fact, Borczuk et al. found airway inflammation in the form of chronic diffuse inflammation in 41% of cases in a series of 68 necropsies in the initial stages of the pandemic.^10^

The third possible explanation for the tracheomalacia in these patients is the presence of previous respiratory diseases. Five patients were affected by asthma, COPD or OSA. Although seldom described, some authors estimate the incidence of tracheomalacia in adult patients with respiratory airway disease to be approximately 12.6%.^6^

The present study has some limitations. Probably, bronchoscopy should be used as a gold‐standard diagnostic tool for this disease,^5^ but CT scan was used in the context of the pandemic, and also in severe stages of the disease in which the use of more invasive techniques was not justified. In fact, emerging evidence in paediatric populations suggests that CT scan can effectively diagnose tracheomalacia and should be considered as a less invasive alternative to bronchoscopy.^11^ Another limitation is that tracheomalacia may also have existed before the SARS‐CoV‐2 infection, especially since most series established the mean age of presentation at 40 years old.^6^ However, in the present series of patients, a guided clinical interview did not reveal any respiratory symptoms previous to the SARS CoV‐2 infection that might have been related to a possible tracheomalacia or any predisposing factor that might explain its presence. Finally, we cannot rule out the development of cicatricial stenosis in the long term.

In conclusion, our results indicate that tracheomalacia is an exceptional sequela of SARS‐CoV‐2 survivors, and is always associated with parenchymal and other airway findings. Nevertheless, the early detection of this condition is crucial in clinical practice. Tracheomalacia causes respiratory symptoms that may have a major impact on patients' lives. It is also a well‐known predisposing factor for repetitive respiratory infections and may entail severe future comorbidity.

## AUTHOR CONTRIBUTIONS

The authors of the manuscript have all contributed significantly to the research, preparation, revision and final production of the manuscript.

## CONFLICT OF INTEREST STATEMENT

The authors have no competing interests to declare in relation to this study.

## ETHICS STATEMENT

The study was approved by the local Ethics Committee, and all the subjects included gave informed consent prior to participation (PR [AG]222/2020).

## Data Availability

The data that support the findings of this study are available on request from the corresponding author. The data are not publicly available due to privacy or ethical restrictions.
